# Post-Traumatic Seizures: A Deep-Dive Into Pathogenesis

**DOI:** 10.7759/cureus.14395

**Published:** 2021-04-10

**Authors:** Fatima Anwer, Federico Oliveri, Fotios Kakargias, Priyanka Panday, Ana P Arcia Franchini, Beshoy Iskander, Pousette Hamid

**Affiliations:** 1 Research, California Institute of Behavioral Neurosciences & Psychology, Fairfield, USA; 2 Cardiology, California Institute of Behavioral Neurosciences & Psychology, Fairfield, USA; 3 Internal Medicine, California Institute of Behavioral Neurosciences & Psychology, Fairfield, USA; 4 Neurology, California Institute of Behavioral Neurosciences & Psychology, Fairfield, USA

**Keywords:** trauma and epilepsy, brain injury and seizure, head injury and epilepsy, post-traumatic epilepsy, motor vehicle accident and epilepsy, post-traumatic seizure

## Abstract

Post-traumatic seizures (PTS) have become an emerging challenge for neurologists worldwide with the rise of brain injuries. Trauma can lead to various outcomes, ranging from naive spasms to debilitating post-traumatic epilepsy (PTE). In this article, we will explore the pathogenesis of convulsions following a concussion. We will look at multiple studies to explain the various structural, metabolic, and inflammatory changes leading to seizures. Additionally, we will explore the association between severity and location of injury and PTE. PTE's pathophysiology is not entirely implicit, and we are still in the dark as to which anti-epileptic drugs will be useful in circumventing these attacks. The purpose of this narrative review is to explain the post-traumatic brain changes in detail so that such attacks can be either thwarted or treated more resourcefully in the future.

## Introduction and background

Traumatic brain injury (TBI) has been emerging as a significant concern recently due to the rising number of cases leading to long-term disability, impacting the healthcare system [1]. TBI can lead to numerous adverse effects ranging from simple seizures to debilitating chronic epilepsy [2].

The risk of developing post-traumatic epilepsy (PTE) varies depending on the site of impact and severity of the injury. It is 4.4% mild TBI, 7.6% for moderate, and 13.6% after severe brain injury [3]. The risk is more in patients with a severe injury, older age, and those suffering from early seizures. Additionally, the risk is also higher among individuals who do not receive anti-epileptic medications [2].

Classification of post-traumatic seizures

**Figure 1:**
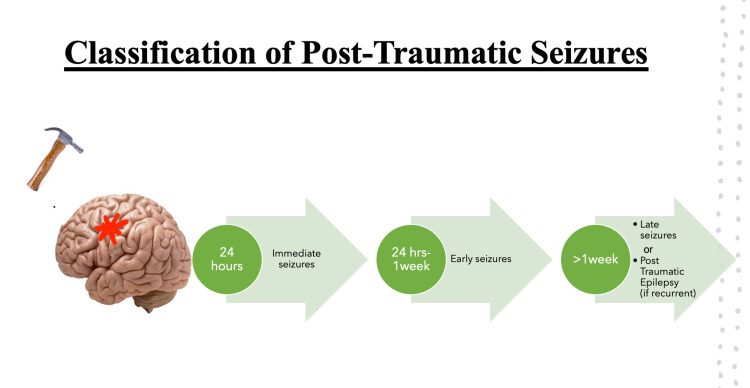
Classification of post-traumatic seizures.

Post-traumatic seizures (PTS) have been classified based on the time of onset after injury (Figure 1). The ones occurring within 24 hours are called "immediate." "Early seizures" appear between 24 hours and one week. Those after one week are "late seizures" [2]. In some cases, repeated unprovoked seizures one week after trauma traditionally referred to as "PTE" might occur. These are different from acute episodes (< 1 week), which are mostly triggered. The PTE can lead to a detrimental effect on the quality of life [4].

Pathogenesis of post-traumatic seizures

Multiple mechanisms have been explained in the past that led to altered brain activity provoking seizures, including increased inflammatory markers, altered blood-brain barrier, changes in astrocytes, and glucose metabolism dysregulation [4-7].

Role of Proteins

Purines play a vital role in the pathogenesis of PTE. The upregulation of urate and decline in adenosine is also a culprit. Additionally, changes in the 2',3' Cyclic adenosine monophosphate pathway lowers the PTE's threshold [8]. Repeated brain injuries, despite the severity, can disturb the normal astrocyte response and cause scar formation. These changes, in turn, decrease Glial fibrillary acid protein expression and downregulate homeostatic proteins [9]. 

The Astrocytes Controversy

The role of astrocytes is still controversial. Some studies show that they protect the healthy brain by forming scar and separating the damaged area. On the other hand, some depict the same fibrosis spot as the focus for the seizure's origin [5,9].

 *Blood-Brain Barrier*

The blood-brain barrier (BBB) guards the neurons against the toxins in the blood and allows very selective molecules to pass through while preventing others. The main component of BBB are endothelial cells with the tight junction and astrocytes end feet and pericytes. Tomkins et al. studied the role of BBB leakiness in PTE. They followed 38 patients for two years after traumatic brain injury and observed the changes in BBB. Those who developed PTE had a greater BBB disruption area than those who recovered without any seizure attacks [10].

Role of Cytokines

Cytokines play a crucial role in the pathogenesis of PTE. Tumour necrosis factor-alpha (TNF-α ) disrupts BBB leading to the entry of leukocytes and causes neuronal degeneration. IL-6 plays a similar part. High mobility group box protein 1 (HMGB1) released by dying cells is also ictogenic. The macrophages, entering the brain after injury, emit chemokine ligand 2 that is ictogenic and causes further damage by recruiting more macrophages [4].

Genetics and Neuroplasticity

Neuroplasticity, the changes in neurons in response to trauma, play an essential role in PTE's pathogenesis, especially temporal lobe epilepsy [11]. Patients who experience epilepsy after injury appear to have poor functionality [12]. Certain genetic factors increase the probability of PTE, like the glutamate transporter gene mutations [13]. In around one-third of the scenarios, PTE is resistant to medication, so a vast understanding of the modifications is vital to reduce PTE occurrence [4].

This narrative analysis attempts to illustrate the changes following damage in the brain. We have obtained evidence from the clinical trials and studies done during the last decade. We used the keywords such as concussion and epilepsy, brain damage and seizure, head injury and epilepsy, post-traumatic epilepsy, motor vehicle crash, and epilepsy, post-traumatic to gather data from the PubMed database. The study aims to explain the particular physiological changes in the body after brain injury and how it reacts to those changes. Trauma can lead to neuronal hyperexcitability and seizures. A thorough understanding of the pathophysiology will decrease the mortality and morbidity following post-traumatic seizures. Thus, minimizing the burden on the healthcare system. Moreover, this will also alleviate the issues that patients and their families face.

## Review

Discussion

The following are the changes that play a vital role in the pathogenesis of PTE.

Post-Traumatic Inflammation

Inflammation plays an exciting role in the pathogenesis of post-traumatic seizures. The three crucial changes that induce convulsions include an increase in HMBP 1, interaction with toll-like receptor-1 (TLR1), interleukin-β (IL-β) interaction with its receptors, and increased tumor growth factor-β (TGF-β) signaling [14].

**Figure 2:**
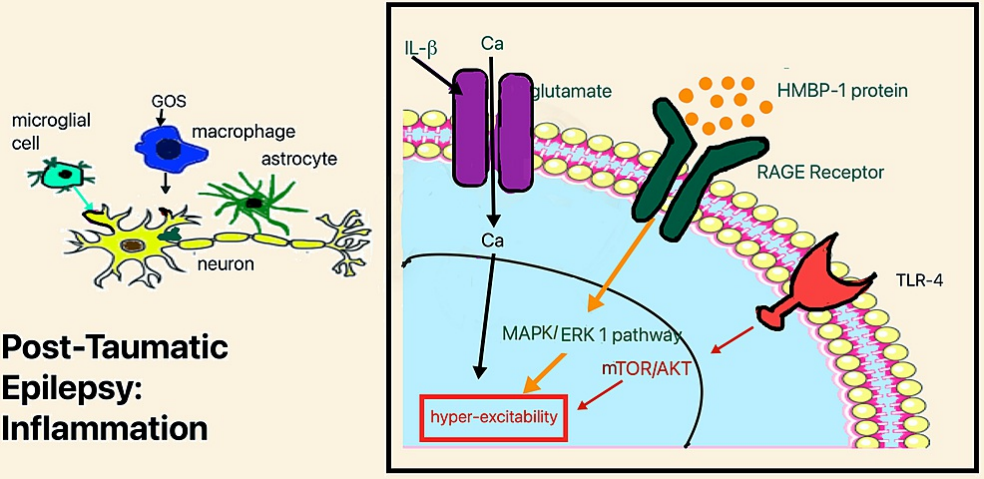
Inflammatory changes after trauma: role of TLR, HMBP1 protein, and IL- β. TLR: toll-like receptor, HMBP1 protein: high-mobility group box 1 protein, IL- β: interleukin-β, MAPK/ERK 1 pathway: mitogen-activated protein kinase/extracellular signal-related kinase 1, RAGE: receptor for advanced glycation end-products, mTOR/AKT: mechanistic target of rapamycin/protein kinase B, GOS: guluronate oligosaccharide

Hippocampus showed increased TLR expression after closed concussion injury (CCI) in the experiment conducted on rodents. The increase TLR causes neurogenesis after trauma [15]. After brain insult, alginate-derived guluronate oligosaccharide (GOS) enters the macrophages. It activates the TLR-4, which in turn causes protein kinase B (Akt) phosphorylation and triggers both nuclear factor-κB (NF-κB) and mechanistic target of rapamycin (mTOR) (Figure 2) [16]. The mTOR pathway, in turn, causes epilepsy. Guo et al. conducted a randomized controlled study by giving animals rapamycin that inhibited the mTOR and studied its subsequent effect. They found that rapamycin decreases PTE's risk, usually occurring ten wks after the primary insult [17].

Prostaglandin E2 (PGE2) expression also increases after injury. Brain permeable compound TG6-10-1 blocks the action of PGE2 and decreases the post-traumatic inflammatory changes and cytokines expression in the brain after injury [18]. Various microglial cells produce IL-β and lead to N-methyl-D-aspartate (NMDA) receptor activation (Figure 2). In turn, NMDA activation causes neuronal hyperexcitability via the glutamate pathway by increasing calcium influx in cells. Hyperexcitability can eventually lead to neurodegeneration [19]. Disruption of BBB allows albumin to enter the brain. Astrocytes also uptake that albumin and, as a result, activate the transforming growth factor-β/activin receptor-like kinase 5 (TGF-β/ALK 5 pathway) [5].

HMBP 1 also causes activation of inflammatory cascade via astrocytes and the transmembrane receptor called receptor for advanced glycation end-products (RAGE). It led to the activation of the mitogen-activated protein kinase/extracellular signal-related kinase 1/2 (MAPK/ERK1/2) pathway (Figure 2) and induced astrocyte COX-2. Pedrazzi et al. blocked RAGE via anti-RAGE antibodies and observed a decrease in inflammation [20].

Structural changes after traumatic brain injury

Traumatic brain injury leads to specific structural changes in the brain. These include the below changes.

BBB Changes

To confirm the changes in BBB after TBI, Tomkins et al. performed a clinical study on 37 post-traumatic patients. All of them had Glasgow Coma Scale (GCS) more than 13 and were seizure-free one week after significant insult. They took images using a Tesla machine to check the BBB integrity and compared them before and after injecting contrast medium Magnetol. They quantified the BBB disruption. Additionally, they documented cortical dysfunction using standard low-resolution brain electromagnetic tomography (sLORETA). They observed slow-wave activity in the brain region with disrupted BBB. They confirmed that the more significant BBB disruption is associated with greater cortical dysfunction and more chances of aberrant slow waves. Subsequently, increasing the risk of PTE [10].

Astrocyte Changes

The astrocytes respond to the injury, trying to seal the damaged portion from the necrotic tissue by forming scar tissue. astrocytes reach the site of injury and create a scar, thus protecting the rest of the tissue. Anderson et al. conducted an interventional study by inhibiting scar formation in rats after spinal cord injury, and thus the cells fail to regenerate. Then they injected some hydrogel depots of growth factors released by astrocytes and documented the scar formation and regeneration of neurons [5,21].

Type of Injury and Seizure

The study on rodents has previously shown changes in the cortex, hippocampus, thalamus, and amygdala associated with an increased risk of Post-traumatic seizures [22]. Tubi et al. recently performed a longitudinal study on humans to check the percentage of trauma patients developing seizures depending on the area of the brain involved. They followed up a cohort of 90 patients for early seizure outcome and the followed 46 patients longitudinally. They performed a prospective cEEG and high-resolution MRI scan to localize lesions. Surprisingly, 75% of the patients who had early seizures (n=24) showed temporal lobe injury on MRI. Out of the 46 patients followed for PTE, 45.7% developed PTE within two years; 85.7% of those who developed PTE also showed hemorrhagic temporal lobe injury on admission. The small study sample and using specifically Asian cohort has added some bias in the study [23].

Lutkenhoff et al. started a prospective study in 2018. They followed 96 patients surviving TBI. They collected imaging data around day 14 after injury and evaluated structural changes in the cortical and subcortical regions. MRI of 57 patients showed subcortical volume loss; 35 had never developed seizures, 14 had early attacks, and eight had late seizure episodes. They also observed cortical ribbon thinning in 46 patients. Among those, 29 remained seizure-free, 10 developed early attacks, and seven showed late seizure activity [24]. Various researchers explain the changes in the brain after injury (Table 1).

**Table 1:** Changes in the brain of patients with seizures and epilepsy after trauma. BBB: blood-brain barrier, PTE: post-traumatic epilepsy

Author	Year of Publication	Study Duration	Population	Sample Size	Outcome
Tomkins et al. [10]	2011	Two years (2005-2011)	Male and female age 27+/-4.2 and 25.3+/- 2.8 years	37	BBB disruption occurred in 82.4% of PTE patients compared to 25% of non-epileptic patients.
Tubi et al. [23]	2019	11 years (Jan 2001- Dec 2011)	Caucasian 73 males 17 females mean age 37.7+/- 17.83 years	90	75% of patients who had early seizures and 85.7% of patients who developed PTE had temporal lobe injury
Lutkenhoff et al. [24]	2020	90 days	Males and females age 6-100 years	96	14 patients who had an early seizure and eight who had PTE had subcortical volume loss 10 patients with early seizures, and seven with late seizures also showed cortical ribbon thinning.

Biochemical and genetic associations with post-traumatic epilepsy

The following are the several biochemical and genetic changes contributing to PTE.

Role of Glycolysis

TBI causes significant metabolic changes, and these changes can be a cause of PTE. Vielhaber et al. demonstrated this in 16 known cases of temporal lobe epilepsy. They used high-resolution respirometry to demonstrate the glucose utilization in the hippocampus. Further, 60-90% of patients during epilepsy showed temporal lobe hypometabolism on [18F] fluorodeoxyglucose positron emission tomography (FDG_PET). The hypometabolism was more in the patients with severe mesial temporal sclerosis [25]. Neural glutamate receptor gene variation was an associated risk factor leading to hyperexcitability after TBI. Of these changes, single nucleotide polymorphism (SNP) in the SLC1A1 gene was most strongly associated with PTE [13].

Increased glucose metabolism after TBI can lead to neuronal hyperexcitability. Koenig et al. conducted a clinical study to support the hypothesis. They utilized the controlled cortical injury CCI model of brain contusion in rats. They used 2-deoxyglucose 2-DG, which is an inhibitor of glycolysis. They first used 2-DG on the cortical brain section in vitro to decrease the epileptiform activity in the brain in mice. During the next step, they treated mice after CCI with 2-DG for one week after injury. Both in vitro and in vivo studies showed a decrease in brain excitability in 2-DG treated rats. Even a ketogenic diet has been tried after TBI to reduce the risk of PTE [26,27].

Changes in Purines and Protein Metabolism

Adenosine is neuroprotective. However, TBI increases the 2',3' cyclic adenosine monophosphate (2',3' cAMP). 2',3' cAMP triggers neuronal apoptosis, necrosis, and autophagy. 2′,3′-cyclic nucleotide 3′-phosphodiesterase (CNPase) metabolizes 2',3' cAMP to 2'-AMP and 3'-AMP which are subsequently degraded to neuroprotective adenosine. 2',3' cAMP and its metabolites decrease lipopolysaccharide-induced TNF-α and CXCL 10 production by activating the A2A-receptor. Adenosine also suppresses TNF-α and CXCL 10 production by microglial cells in the brain. Determining the protein biomarkers raised after TBI can also help understand the pathogenesis and prevention of PTE [8,28,29].

Newell-Rogers et al. explained the role of astrocytes and peripheral lymphocytes activation in epileptogenesis. Macrophages cause neuroinflammation, which plays a vital role in the pathogenesis of PTE. The cytokine macrophage migration inhibitory factor (MIF) increases in the brain after TBI. MIF can affect the hippocampus neuronal activity, lowering the threshold of PTE. Inhibiting MIF interaction with the cluster of differentiation 74 (CD 74) by using MIF antagonist (S, R)3-(4-hydroxyphenyl)-4,5-dihydro-5-isoxazole acetic acid methyl ester (ISO1) resulted in decreased astrocytosis. However, ISO1 couldn't prevent brain degeneration. The Fluid Percussion injury model was used in mice and injected ISO 1 30 minutes after an injury to observe the results. Moreover, to explain the role of ISO 1 in gut lymphocytes activation they, checked the γδ T cells in the gut after trauma. They compared before and after levels of γδ T cells after ISO1 injection. The γδ T cells were low after ISO1 therapy [30].

Post cranioplasty seizures

Decompression (DC) surgery lowers the raised Intracranial pressure after trauma, and cranioplasty reconstructs the brain. Cranioplasty also lowers the threshold of convulsions. Spencer et al. conducted a systematic review. It compared eight previous studies, including 919 cranioplasty patients. They established the link between various factors leading to post craniotomy seizures (Table 2). The pooled incidence of post-cranioplasty seizures was 5.1% with 95% CI ( 2.6-8.2) [31].

**Table 2:** Factors affecting post-cranioplasty seizures. DC: decompression

Risk Factor	Odds Ratio (95% CI)	P-Value
Increasing age	6.1	0.006
Contusion at cranioplasty site	4.8	0.015
Use of monopolar diathermy at cranioplasty	3.5	0.04
High DC- cranioplasty interval	-	0.06

Limitations

The post-traumatic events leading to seizures include inflammation by cytokines and astrocytes. The role of astrocytes is still controversial and needs to be studied. Most of the studies mentioned in this narrative review are animal-based. Tubi et al. used a small Caucasian cohort in their clinical trial. The high attrition rate might have caused the bias [23].

## Conclusions

Cases of brain injuries are continually growing with the increase in road traffic accidents. Trauma can lead to various outcomes from simple seizures and debilitating post-traumatic epilepsy. Multiple changes occur after a head injury that can lead to these attacks. Researchers globally are actively searching for new ways to explore the events leading to these convulsions. The inflammatory changes that cause PTE and seizures include upregulation of the mTOR pathway via TLR-1. HMBP-1 protein activates the MAPK/ERK 1/2 pathway. NMDA receptor activation increases neuronal hyperexcitability. Systemic changes following trauma include BBB disturbance, cortical volume reduction. Temporal lobe injury is more associated with convulsions than other parts of the brain. The glutamate receptors gene mutation also lowers the threshold of PTE. The increased glucose metabolism after trauma also leads to neuronal hyperexcitability. Adenosine is neuroprotective, which may prevent inflammation as nerves lose neuroprotective adenosine after trauma.

Despite all the research, the pathogenesis of seizures after trauma is still not completely understood. Some people have crippling chronic epilepsy, while others even lose their lives due to acute convulsions. No known anti-epileptic drug is there that can be safely relied on to prevent or treat these episodes. Most of the studies we discussed in this narrative review are animal-based. Randomized controlled trials, placebo-controlled with a large human population, should be conducted to determine the PTE's exact cause.
